# Long-term prognosis of surgical treatment for early ampullary cancers and implications for local ampullectomy

**DOI:** 10.1186/s12893-015-0019-z

**Published:** 2015-03-22

**Authors:** Junmin Song, Hongxiang Liu, Zhen Li, Chao Yang, Yuling Sun, Chaojie Wang

**Affiliations:** Department of General Surgery, The First Affiliated Hospital of Zhengzhou University, Zhengzhou, China; Department of Oncology, Henan Provincial People’s Hospital, Zhengzhou, China

**Keywords:** Ampullary cancer, pT1 stage, Local ampullectomy, Lymph node metastasis

## Abstract

**Background:**

Early ampullary cancers present with good prognosis. Pancreaticoduodenectomy (PD) has been standard treatment for ampullary cancers, but it remains high rate of postoperative complications. So there raises a discussion on the role of local ampullectomy for early ampullary cancers (mainly focusing on pT1).

**Methods:**

89 patients with pT1 ampullary cancer who underwent surgical treatment between 1978 and 2010 were retrospectively studied.

**Results:**

Rate of postoperative complications, especially post-operative pancreatic fistula (*P* = 0.009), after PD was higher than after local ampullectomy, . Multivariate analysis showed that tumor size (HR 2.204; *P* = 0.014), lymph node metastasis (HR 4.362; *P* < 0.001), lymph vascular invasion (HR 4.258; *P* < 0.001), and perineural invasion (HR 4.467; *P* < 0.001), gross morphology (HR 2.536; *P* = 0.004) and tumor grade (HR 4.213; *P* = 0.001) were independent risk factors for long-term survival, as well as risk factors for failure of ampullectomy in early ampullary cancer. For patients absent of these factors, local ampullectomy would achieve a good prognosis.

**Conclusions:**

Because of high rate of lymph node metastasis, PD should be preferably performed for radical resection. Local ampullectomy could be an alternative for patients in high operative risk; and would achieve a good outcome in patients whose tumors were well differentiated and showed polypoid gross morphology and size ≤1 cm.

## Background

Ampullary cancer was the second common peri-ampullary malignancy. Recent literatures reported a 5-year survival rate ranging from 32% to 65%.[1-5] Pancreatoduodenectomy (PD), the standard surgical strategy for ampullary cancer, was still associated with high rate of postoperative complications, reaching to 33%-52%.[5,6] Therefore, local ampullectomy had been attempted to be an alternative to PD for early cancer .

Local ampullectomy, first described by Halsted in 1899, was generally accepted in treatment of small benign tumors; but controversy still remained about expanding the indications to early ampullary cancers (mainly focusing on pT1) because of the high rate of recurrence.[6-10] Furthermore, because of the limited number of ampullary cancer patients, as regards to the surgical mode of early ampullary cancers, indications for performing local ampullectomy were not very clear and well-accepted.

In this study, we collected patients with pT1 ampullary cancers who underwent a surgical treatment, including PD and local ampullectomy; and further analysis were done to determine the feasibility of local ampullectomy.

## Methods

### Patients

There were 89 patients with a pT1 ampullary adenocarcinoma who underwent surgical resection at the First Affiliated Hospital of Zhengzhou University between January, 1978 and December, 2010. Carcinomas of the distal bile duct, pancreas, or duodenum, as well as carcinoid tumors of the ampulla, were excluded. The pT1 stage meant that according to the seventh edition of the American Joint Committee on Cancer (AJCC), the tumor was confined to ampulla of Vater, As present study sought to examine the outcomes following surgical management of ampullary cancers, patients who had endoscopic excision of ampullary neoplasm were also excluded from this study. All the patients did not receive adjuvant therapy. The following data were collected: demographics, operation details, postoperative complications, tumor size, lymph node metastasis, lymphovascular invasion. Specific complications such as pancreatic fistula and delayed gastric emptying were defined according to the International Study Group of Pancreatic Surgery definition.[11,12] This research was approved by the Ethics Committee of the First Affiliated Hospital of Zhengzhou University.

### Pre-operative staging

All the patients had a magnetic resonance (MR) examination before operation. Endoscopic ultrasound (EUS) was used to determine the stage of 82 patients after 1990.

### Operative approach

Following were rules for choosing operation approach: 1. If the tumor size was >4 cm, PD would be performed. 2. For patients with enlarged abdominal lymph node identified by pre-operative imaging examination, PD would be performed. 3. For patients who were older than 70 years and refused to receive PD, local ampullectomy was performed.

The technique of pancreaticoduodenectomy was performed as previously described.[2,11] Drains were routinely placed intraoperatively near the pancreatic and biliary anastomoses. Local ampullectomy consisted of local resection of the ampulla through a transduodenal approach followed by a pancreaticobiliary sphincteroplasty.[9] Lymphadenectomy was not conducted.

### Statistical analysis

Categorical variables were compared using *Fisher’s* exact test. Continuous variables were compared using the Mann–Whitney sum test. Actuarial survival was estimated using the non-parametric product limit method (e.g. *Kaplan–Meier*) and differences in survival were examined by the log-rank test. Multivariate Cox proportional hazard models were employed to determine clinicopathological factors which were associated with long-term survival. The most parsimonious model was created using a step-wise approach, which included factors with statistically significance (e.g.*P* ≤ 0.10) in univariate analysis. Averages were provided as median values and statistical significance was designated as *P* < 0.05. All statistical analysis were performed using SPSS 17.0 software (SPSS Inc. Chicago, IL, USA).

## Results

### Pre-operative staging

As indicated in Table 1, the accuracy of EUS in assessing the depth of carcinoma extension was 87.8% (72/82), superior to the MR (67.0%, *P* < 0.001); The results of EUS in N staging of ampullary carcinomas were 65.9% (54/82), inferior to the MR (80.90%, *P* = 0.024).

**Table 1:** **Accuracy of EUS, MR in the detection and staging of ampullary tumors**

	**EUS**	**MR**	***P***
T staging	87.8% (72/82)	70.8% (63/89)	0.001
Overstaging	9.8% (8/82)	12.4% (11/89)	
Understaging	2.4% (2/82)	22.5% (20/89)	
N staging	65.9% (54/82)	85.4% (76/89)	0.024
Overstaging	23.1% (19/82)	14.6% (13/89)	
Understaging	11.0% (9/82)	5.6% (5/89)	

### Surgical details and post-operative complications

Among the 89 patients with pT1 ampullary cancer who underwent surgical resection, PD were performed in 63 (70.7%) patients, while local ampullectomy were did in 26 (29.2%) patients. Intraoperative and postoperative data were summarized in Table 2. Mean blood loss was higher in patients who underwent PD than in patients who underwent local ampullectomy (281.28 ml vs 97 ml; *P* < 0.001). Similarly, PD took longer operative time than local ampullectomy (307.16 min vs 214.46 min; *P* < 0.001). As expected, patients with PD had more post-operative complications than patients with local ampullectomy. There were 3 in-hospital deaths caused by postoperative complications in patients with PD, while there were no in-hospital death in patients with local ampullectomy. Post-operative pancreatic fistula occurred in 21 patients with PD and in 2 patients with local ampullectomy (*P* = 0.009). Other complications, such as wound infection, delayed gastric emptying, bile leakage and pleural effusion showed no significant differences between the patients with PD and with local ampullectomy.

**Table 2:** **Intra- and postoperative data on patients undergoing resection of ampullary cancers stratified by procedure type**

	**Local ampullectomy (** ***n*** **= 26)**	**Pancreaticodudenectomy (** ***n*** **= 63)**	***P***
Blood lost, mean ± SD (ml)	97.16 ± 36.87	281.28 ± 201.18	<0.001
Intra-operative transsfusion	0 (0.0%)	10 (15.9%)	<0.001
Operation time,mean ± SD (min)	214.46 ± 23.76	307.16 ± 28.08	<0.001
POPF	2 (7.6%)	21 (33.3%)	0.009
Intra-abdominal infection	8 (30.7%)	18 (28.6%)	0.513
Abdominal abscess formation	0 (0.0%)	7 (11.1%)	0.080
Anastomotic leakage			
Bile leakage	1 (3.8%)	5 (7.9%)	0.431
GJ anastomosis leakage	_	2	_
DGE	0 (0.0%)	5 (7.9%)	0.169
Wound infection	1 (3.8%)	3 (4.8%)	0.667
Pleural effusion	0 (0.0%)	3 (4.8%)	0.350
Reoperation	0 (0.0%)	5 (7.9%)	0.169
In-hospital death	0 (0.0%)	3 (4.8%)	0.350

POPF: postoperative pancreatic fistula; GJ: gastrojejunostomy; DGE: delayed gastric emptying.

### Clinicopathological characteristics

As illustrated in Table 3, of the 89 patients inclued in this study, 42 are males and 47 are females; Several clinicopathological parameters such as jaundice (local ampullectomy vs PD, 2/26 vs 31/63, *P* < 0.001), gross morphology (local ampullectomy vs PD, 24/26 vs 38/63, *P* = 0.002) and tumor size (local ampullectomy vs PD, 7/26 vs 33/63, *P* = 0.024) shows statistically significant differences between the patients with PD and with local ampllectomy, while other clinicopathological parameters, such as, age, gender, tumor grade shows no. In addition, because lymph node and perineural dissection was not performed in ampullectomy, it is nonsense to compare the difference in lymph node metastasis, lymphovascular invasion and perineural invasion between the two groups.

**Table 3:** **Patient Characteristics of 89 PatientsWith pT1 Ampullary Cancer**

	**Local ampullectomy (** ***n*** **= 26)**	**Pancreaticoduodenectomy (** ***n*** **= 63)**	***P***
Age, mean ± SD	66.25 ± 14.149	67.16 ± 11.658	0.942
Gender			0.543
Female	14	33	
Male	12	30	
Jaundice			<0.001
Positive	2	31	
Negative	24	32	
Gross morphology			0.002
Polypoid	24	38	
Ulcerative	2	25	
Tumor size			0.024
≤1 cm	7	33	
>1 cm	19	30	
Tumor grade			0.128
Well	10	15	
Moderate/Poor	16	48	
Lymph node metastasis			
Positive		22	
Negative	___	41	___
Lymphovascular invasion			
Positive	___	25 38	___
Negative			
Perineural invasion			
Positive		6	
Negative	___	57	___

### Survival analysis

**Figure 1:**
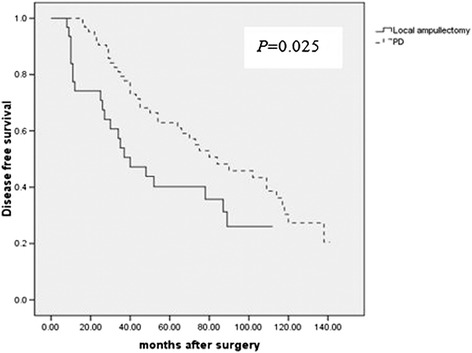
**Patients in local ampullary group suffered from a poor disease-free survival than in PD group.**

A number of clinicopathological factors were associated with survival in the patients with ampullary cancers,. Univariate analysis showed that gross morphology, tumor size, tumor grade, lymph node metastasis and lymphovascular invasion were significant predictors of poor survival. (Table 4) The overall survival showed no significant difference between patients with PD and with local ampullectomy. But patients with PD had longer disease-free survival. (Figure 1) Of patients with local ampullectomy, 10 patients were found to have recurrence within two year after surgery (2 in pancreatic, 6 in peri-pancreatic lymph nodes, 2 in liver). No recurrence was found in patients with PD. Multivariate analysis showed that tumor size, lymph node metastasis, lymphovascular invasion, grass morphology and tumor grade turned out to be independent risk factors which influenced patients’ survival (Table 4).

**Table 4:** **Survival analysis for 89 Patients With pT1 Ampullary Cancer**

	***Univariate analysis***			***Multivariate analysis***	
	**5-year survival rate**	**10-year survival rate**	***P***	***HR (95%CI)***	
Age			0.756	______	
<65	69.0%	44.1%			
≥65	67.0%	41.0%			
Gender			0.880	______	
Female	69.3%	43.3%			
Male	67.1%	40.6%			
Jaundice			0.683	______	
Positive	59.1%	39.8%			
Negative	68.3%	49.1%			
Gross morphology			0.006	1 2.536 (1.351-4.860)	0.004
Polypoid	75.8%	48.0%			
Ulcerative	44.2%	29.5%			
Tumor size			0.016	1 2.204 (1.171-4.148)	0.014
≤1 cm	78.8%	50.9%			
>1 cm	54.7%	37.7%			
Tumor grade			0.017	1 4.213 (1.868-9.504)	0.001
Well	88.1%	61.1%			
Moderate/poor	56.8%	34.6%			
Lymph node metastasis			<0.001	1 4.362 (2.126-8.952)	<0.001
Negative	74.4%	49.4%			
Positive	32.1%	0.0%			
Lymphovascular invasion			<0.001		<0.001
Negative	69.4%	51.l%		1	
Positive	33.2%	12.1%		4.258 (2.013-8.861)	
Perineural invasion			<0.001		<0.001
Negative	72.1%	45.6%		1	
Positive	36.8%	15.3%		4.467 (2.315-9.034)	
Operation style			0.639		0.549
Pancreaticoduodenectomy	65.6%	38.7%		1	
Local ampullectomy	64.6%	42.6%		1.372 (0.854-1.762)

### Predictability of lymph node metastasis

Of the above prognostic factors, lymph node metastasis was the key predictive factor that made local ampullectomy inappropriate. Therefore, we investigated the relationship between lymph node metastasis and other prognostic factors. As shown in Table 5, lymph node metastasis was identified in 22 (34.92%) of 63 patients who underwent PD; Patients with well-differentiated tumors showed a lower rate of positive lymph nodes metastasis (8%) than patients with moderate/poorly differentiated tumors (31.25%) (*P* = 0.022); In regard to tumor size, the patients with tumor size ≤1 cm showed a lower rate of positive lymph nodes metastasis than patients with tumor size >1 cm (15% vs 32.65%, respectively, *P* = 0.046); Similarly, lymph nodes metastasis tended to occur more frequently in patients with ulcerative morphology than patients with polypoid morphology (40.74% vs17.74%, respectively, *P* = 0.021). So tumor grade, morphology and size significantly correlated with lymph node metastasis (*P* < 0.05).

**Table 5:** **Correlation between pathological characteristics and lymph node metastasis**

	**Number of patients with lymph node metastasis**	***P***
Gross morphology		0.021
Polypoid	11/62 (17.74%)	
Ulcerative	11/27 (40.74%)	
Tumor size		0.046
≤1 cm	6/40 (15.00%)	
>1 cm	16/49 (32.65%)	
Tumor grade		0.022
Well	2/25 (8.00%)	
Moderate/Poor	20/64 (31.25%)	

### Correlation between the risk factors of failure after ampullectomy and the indication factors of ampullectomy

As shown in Table 5, tumor size, tumor grade, and gross morphology were risk factors for ampullectomy in early ampullary cancer. In these patients, 4 patients absent of all these 3 risk factors, showed a survival of 57, 107,111, 142 months, respectively. Therefore, absence of these risk factors might be the indications for local ampullectomy.

## Discussion

Early ampullary cancers limited to the ampulla of Vater (pT1) showed a fairly good prognosis. These tumors could be radically removed in all cases and showed a 5-year survival rate of 60% to 90% according to the previous reports.[5,13,14] In order to achieve such a good outcome, complete resection of the tumor was mandatory.[15,16].

There was no doubt that the standard operation for ampullary cancer should be PD. But PD showed high rate of postoperative complications, so local ampullectomy had been an alternative for early ampullary cancer and showed comparable survival as PD [17-20]. In this study, we identified patients with pT1 ampullary cancer treated with local ampullectomy and standard PD, and analysed the clinical data of these patients. The results showed that patients treated with local ampullectomy showed overall survival equal to those patients treated with PD; but the disease-free survival time turned to be shorter in patients treated with local ampullectomy than that in patients treated with PD. Among the clinicopathological factors, lymph node metastasis was the main one which caused ampullectomy fail.

Therefore, the indications for local ampullectomy treatment should and must be fully explained and strictly executed. Botsios [18] et al. recommended ampullectomy for T1 cancer and Yoon et al. [5] recommended it for pTis cancers or pT1 cancers with size≦ 1.0 cm in patients with a high operative risk. However, because of lack of a large multi-center clinical studies, a generally accepted standard was still lacking.

Various of investigations were available for staging ampullary tumors, including CT scanning, magnetic resonance (MR) imaging, endoscopic ultrasound (EUS) and transpapillary intraductal ultrasound. Although EUS could accurately define the depth of invasion in 75% to 83% of cases, the reported accuracy of EUS for detecting lymph node metastasis ranged from 54% to 68%. [21-23] CT scanning had been compared with EUS in staging utility in a number of studies and shown a lower agreement for T and N staging with the histopathology when compared with EUS.[23-25] MR imaging had been reported that it harbored a sensitivity of 46% to 93.3% [26-28] in T staging; and one study presented a sensitivity of 77% for nodal detection [26]. In these patients included in this study, EUS was superior to MR in T staging, and achieved an accuracy of 87.8%; and only 2.4% patients were under-staged. So, EUS was strongly recommended before operation.

Previous studies showed that lymph nodes metastasis was associated with tumor size and tumor grade. Bottger and Junginger [29] reported that lymph node metastases was not found in tumors smaller than 0.6 cm, or in well-differentiated tumors; so local ampullectomy for ampullary cancer might be used in such cases. Brown [30] reported 10 cases of pT1 ampullary cancers with no lymph node metastasis. Winter et al. [6] also reported that tumor size ≥ 1 c m, poor histological grade, perineural invasion, microscopic vessel invasion and T stage were significantly associated with lymph node metastasis. Lymph node metastases were present in nearly 30% of patients with T1 diseases. In present study, we explored the relationship between lymph node metastasis and the clinicalpathological factors. The result showed that patients with pT1 cancers had a 34.92% (22/63) rate of lymph node metastasis. Rate of lymph node metastasis in patients with well-differentiated tumors was 8%,; In patients with tumor size ≤1 cm it was 15% and in patients with ulcerative tumor morphology it was 40.74%. This meant that well-differentiated tumors with polypoid gross morphology and size≦1 cm might be the indications of local ampullectomy in high operative risk patients.

However, there were some disadvantages in our study. Firstly, this study was retrospectively conducted, resulting in less strong evidence. Secondly, this was a single center study, the number of patients were limited. Therefore, a large scale, multi-center RCT study should be performed in the future to verify the feasibility of local ampullectomy in high risk patients.

## Conclusions

Because of high rate of lymphovascular invasion, PD should be preferably performed for radical resection. Local ampullectomy could be an alternative in high operative risk patients and could achieve a good outcome when the tumors were well differentiated and showed polypoid gross morphology and size ≤1 cm.
